# Relationships between leader behaviors, employee well-being, and profit target achievement: evidence from a company-wide survey in Japan

**DOI:** 10.1038/s41598-026-51197-4

**Published:** 2026-05-16

**Authors:** Masato Kanai, Yukiko Uchida, Arata Yuminaga, Keiko Mizuno, Gakuse Hoshina, Nobuo Sayama

**Affiliations:** 1 Kyoto University Institute for the Future of Human Society, Yoshida-shimoadachicho, Sakyo-ku, 606-8304 Kyoto-shi Japan; 2 Accenture Japan Ltd., Akasaka Intercity AIR, 1-8-1 Akasaka, Minato-ku, 107-8672 Tokyo Japan; 3 Integral Corporation, GranTokyo South Tower, 1-9-2, Marunouchi, Chiyoda-ku, 100-6610 Tokyo Japan; 4 Graduate School of Management, Kyoto University, Yoshida-honmachi, Sakyo-ku, 606-8501, Kyoto-shi, Japan

**Keywords:** Leader behaviors, Employee well-being, Happiness at work, Financial performance, Profit target achievement, Business and management, Business and management, Psychology, Psychology

## Abstract

What kinds of leader behaviors can simultaneously promote employee well-being and financial performance? Sustained organizational success requires advancing both outcomes, yet how leadership behaviors are associated with these outcomes remain unclear. Using four consecutive years of company-wide survey data from a Japanese homebuilder, we examined relationships between nine leader behaviors and branch-level employee well-being, as well as branch-level financial performance. After confirming the factor structure of the behavior items, we conducted hierarchical linear models and cross-lagged panel models. We found that three types of leader behaviors (employee development, communication, and sustainable work efficiency) were positively related to branch-level employee well-being, after controlling for branch demographics and year effects. In contrast, one behavior (workplace strategy, defined as clarifying the branch strategy and individual goals) was positively related to financial performance. In the longitudinal analyses, leader behaviors were not associated with subsequent employee well-being, whereas higher employee well-being was consistently associated with higher subsequent ratings of leader behaviors. We discussed priorities for allocating limited leadership resources to foster a happy workplace and contribute to financial outcomes, as well as the potential mechanisms underlying the observed associations between leader behaviors and both outcomes.

## Introduction

Improving operating profit and enhancing employee well-being are important issues for many companies and pursuing both in parallel is essential for the sustained growth of Japanese firms. In Japan, intensifying global competition and rising cost pressures have made the improvement of operating profit margins a major issue for many companies. According to a survey by the Bank of Japan, 60–80% of responding companies responded that their business conditions were “not so good” or “bad”^1^. Other surveys of Japanese companies also showed that “improvement of profitability” has been recognized as one of the top management issues for five consecutive years^2, 3, 4^.

In addition, growing attention has been directed toward employee well-being, with the view that fostering engaged and purposeful employees can in turn contribute to enhanced organizational performance. Indeed, many studies have shown positive relationships between job satisfaction and job performance^5^, and a meta-analysis has also shown that overall employee satisfaction and engagement are positively related to profit margins^6^. These findings suggest that improving well-being is not merely “welfare” but a strategic investment that underpins corporate performance itself.

There are multiple definitions of employee happiness and well-being; however, in recent years, there has been a growing tendency to regard them as multidimensional concepts that go beyond “job satisfaction.” For example, happiness at work has been discussed as “an umbrella concept that includes a large number of constructs ranging from transient moods and emotions to relatively stable attitudes and highly stable individual dispositions at the person level to aggregate attitudes at the unit level”^7^. A study on Japanese companies also provided an overview of research on employee well-being and adopted an approach to measure well-being within a comprehensive framework that encompasses job satisfaction, organizational commitment, life satisfaction, eudaimonic happiness, and interdependent happiness^8^. There are also many studies that examine employee well-being from multiple perspectives^9, 10^, and based on this line of research, employee well-being should be regarded as a broader concept that is not limited to job satisfaction. Yet, despite this broadened perspective, the question of how organizations can actually foster employee well-being remains largely unresolved. One of the research questions of this study is to explore this issue, with a particular focus on leader behaviors as a potential key factor.

Building on prior research, leader behavior is considered a key factor in promoting employee well-being and corporate profitability. Since the 1950s, many researchers have focused on leaders’ behaviors and sought to classify them. These efforts have repeatedly conceptualized leader behaviors in terms of task accomplishment (e.g., goal setting and progress monitoring) and group maintenance (e.g., respecting opinions and providing praise)^11^, and meta-analyses have shown that these categories are related to both employee well-being and organizational performance. For example, the terms *initiating structure* and *consideration* were introduced to represent task accomplishment and group maintenance^12^, and a meta-analysis demonstrated that these behaviors enhance subordinates’ job satisfaction and group performance^13^. Other frameworks include the distinction between *job-centered* and *employee-centered* supervision^14^, the *Performance* and *Maintenance* functions in PM theory^15^, and the *production-oriented* and *people-oriented* dimensions in the Managerial Grid^16^. A meta-analysis reported that task behaviors and relationship behaviors were positively related to development speed and sales performance^17^. Since the 2000s, several studies have extended these classifications by adding *change-oriented behavior* as a third dimension^18, 19^. Reviews have further argued that change-oriented and relationship-oriented behaviors help reduce job stress and support psychological health^20^.

However, research on leader behaviors still faces certain challenges, one of which is the need to examine leader behaviors with sufficient differentiation. For example, it was noted that many traditional leadership studies have relied on only one or two broad meta-categories, such as task accomplishment and group maintenance, which was identified as a problem^21^. Indeed, in business practice, practical guidance that can be immediately implemented is often sought, rather than abstract theories. An approach that decomposes leader behaviors as argued in previous research^21^ and identifies critical actions by comparing their relations with outcomes can provide concrete implications for understanding effective leadership and for designing leadership training and development programs. To address this issue, Yukl and colleagues proposed a hierarchical taxonomy that positioned 11 narrowly defined specific behaviors under three broader behavior meta-categories. Moreover, in the same article, the effects of each specific behavior on subordinates’ motivation and performance were tested, and the usefulness of differentiating behaviors was emphasized.

Another challenge is the need to examine the effects of leader behaviors on performance using objective indicators, rather than relying mainly on subjective evaluations or experimental tasks. However, such data are scarce. In other words, only a limited number of studies have investigated the effects of leader behaviors using actual financial indicators, such as sales and profits, as measures of firm performance. For example, indicators of team productivity/quantity and group performance aggregated in previous meta-analyses included diverse measures such as the number of items produced in simulation games or the speed of product development, while studies employing actual financial indicators accounted for only a small portion of the data collected in these articles^13, 22^. Another meta-analysis classified team performance indicators into subjective ratings provided by team leaders or members and objective performance such as development speed or daily sales. However, detailed analyses of objective performance was abandoned due to limited data^17^. This tendency is also evident in leadership research on Japanese firms, where studies employing financial indicators as outcome measures remain limited^23, 24^. Considering the possibility that subjective evaluations and experimental task performance may not necessarily align with changes in actual financial indicators, it is important to accumulate evidence on the effects of leader behaviors using actual financial indicators as objective outcomes. Moreover, performance indicators such as sales and profits are strongly influenced by differences in industry characteristics, business models, and firm size. Therefore, when data are collected across multiple companies, it becomes difficult to accurately isolate the true effects of leader behaviors on performance. By contrast, analyzing branches within a single firm minimizes such confounding influences and enables more valid comparisons, as the units share comparable organizational systems and market environments. To address this methodological limitation, the present study examines branch-level financial performance within a Japanese homebuilder, where profit indicators can be directly compared across branches.

Furthermore, longitudinal studies that examine the effects of leader behaviors on employee well-being and financial performance as sustained processes over time remain limited^25, 26^. Some studies have reported that, in relation to well-being, abusive supervision and directive or empowering leadership exhibit reciprocal cross-lagged effects with subordinates’ emotional exhaustion, job neglect, fatigue, and engagement^27, 28^. In relation to performance, charismatic leadership has been shown to demonstrate reciprocal cross-lagged effects between subjective performance and objective financial indicators^29^. These studies suggest that leader behaviors and various outcomes are characterized by bidirectional causal relationships, and that over time they reinforce each other, gradually creating desirable states through a cyclical process. However, many empirical studies have examined only cross-sectional relations or short-term effects. This highlights the need for longitudinal analyses designed to address bidirectionality and to clarify leader behaviors and processes that enhance employee well-being and financial performance over time.

Building on the above discussion, this study uses large-scale data from Japanese firms to clarify the relationships between fine-grained leader behaviors, employee well-being, and profit target achievement rate through multivariate analyses. First, we cross-sectionally examine how evaluation scores of leader behaviors, assessed at a finer-grained level than broad meta-categories such as *task-oriented behaviors* and *relationship-oriented behaviors*, contribute to employee well-being and profit target achievement rate. Then, based on these results, we identify candidate leader behaviors for longitudinal analysis. Subsequently, using longitudinal models applied to panel data, we investigate the processes through which these leader behaviors sustainably enhance both well-being and financial performance over time.

## Method

### Participants and procedure

This study used four years (2020–2023) of company-wide survey data, financial records, and branch-level background information (specifically, the number of employees and the average tenure of branch members) from Company A, a Japanese homebuilder. The survey was administered annually to all employees to assess leader behaviors and well-being. For the main analyses, we used branch-level averages of employee responses from the single-family and multifamily housing divisions, restricted to branches that could be matched with performance data for the same year. Table 1 shows the number of branches analyzed, the number of employees with identifiable branch affiliation, and their demographic characteristics.

This research was a secondary data analysis study based on previously collected data. Company A provided these data to the authors after obtaining internal approvals for academic use under university research, and this data sharing is covered by the exception for third-party provision for academic research purposes set forth in Article 27(1)(vii) of Japan’s Act on the Protection of Personal Information. All datasets provided to the authors were anonymized, and no identifiable information was included. The conduct of this study was reviewed and approved by the ethics review board of Kyoto University Psychological Unit (approval number: 26-P-16). All procedures adhered to the Declaration of Helsinki and relevant guidelines and regulations.

**Table 1:** Summary of analyzed branches, identified employees, and demographic characteristics.

Year	Total branches	Single-family division branches	Multifamily division branches	Number of employees	Male	Female	Average age (M ± SD)
2020	106	75	31	10,670	7836	2834	40.64 ± 12.37
2021	103	71	32	10,562	7679	2883	40.96 ± 12.52
2022	101	69	32	10,583	7635	2948	41.29 ± 12.79
2023	98	65	33	10,774	7774	3000	41.67 ± 13.16

### Measures

#### Leader behaviors

Regular and contract employees belonging to branches in the single-family and multifamily divisions evaluated the quality of their branch managers’ practices, under the instruction to keep in mind how their leaders managed the presented aspects. Responses were collected using a five-point Likert-type scale (“strongly disagree”, “slightly disagree”, “neither agree nor disagree”, “almost agree”, and “strongly agree”), scored as 0, 25, 50, 75, and 100 points, respectively. Company A provided four years of data (2020–2023). However, individual-level responses were available only for 2022 and 2023 because the 2020 and 2021 datasets, which contained branch-level averages and information identifying each employee’s branch affiliation, lacked individual identifiers. The measure consisted of 28 items developed by Company A. The items were categorized into nine domains based on the company’s framework (Table 2A): corporate strategy (α = 0.90), environmental initiatives (α = 0.87), workplace strategy (α = 0.92), communication (α = 0.90), employee development (α = 0.90), diversity (α = 0.84), legal compliance (α = 0.90), human rights (α = 0.88), and sustainable work efficiency (α = 0.87). These Cronbach’s α coefficients were estimated using the individual-level data from 2022 to 2023. Only the legal compliance domain contained four items, whereas all other domains contained three items each. For 2020–2021, we used branch-level mean scores provided by Company A, whereas for 2022–2023, we calculated branch-level means from individual employee responses. The response rate among employees whose branch affiliation could be identified ranged from 72.55% to 84.30% per year, with an average of 79.36% across the four years.

#### Employee well-being

The same groups of employees responded to the *Well-Being Circle*^30^, from which we used the overall and workplace well-being scores for the analyses (Table 2B). Company A provided individual-level data for all four years (2020–2023). The overall well-being score (α = 0.77) was calculated as an aggregate of eight items on life satisfaction and eight items on positive emotions experienced during the past two weeks. The workplace well-being score (α = 0.90) was calculated from ten items, which were categorized into four aspects: a safe and secure culture, an atmosphere of a trusted workplace, an atmosphere that encourages challenge, and workplace recommendation degree. The Cronbach’s α coefficients were estimated using individual-level responses from all four years. Both scores were assessed by employees about themselves, using a seven-point Likert-type scale (“strongly disagree”, “disagree”, “slightly disagree”, “neither agree nor disagree”, “slightly agree”, “agree”, and “strongly agree”), and were transformed into a 100-point scale. For the analyses, branch-level averages of these scores were calculated from individual employee responses for all years. The response rate among employees whose branch affiliation could be identified ranged from 83.76% to 89.08% each year, with an average of 86.88% across the four years.

**Table 2:** Meaning of each variable and examples of items.

Variables	Meanings	Examples of items
A. Leader behaviors		
Corporate strategy	Whether company-wide strategies are sufficiently shared	In the workplace, the content and policies of company-wide meetings are communicated and shared.
Environmental initiatives	Whether Company A’s environmental initiatives are understood and practiced	In the workplace, the “[Masked term]” that the company has made to society is understood and practiced.
Workplace strategy	Whether workplace strategies as well as individual goals and roles are understood	In the workplace, workplace strategies and vision are clearly articulated.
Communication	Whether the workplace is cooperative, communicative, and open to discussion	In the workplace, communication is good, and there is a culture in which the necessary information for work is communicated.
Employee development	Whether individual growth is valued and supported	In the workplace, there is a culture of developing human resources through appropriate instructions and advice, supportive assistance, and timely feedback.
Diversity	Whether diversity, women’s participation, and the balance between work and family responsibilities are valued	In the workplace, there is a culture of respecting diversity and applying it to work.
Legal compliance	Whether compliance and transparency are maintained	In the workplace, a system for checking compliance is in place, enabling smooth work operations.
Human rights	Whether the workplace does not tolerate harassment or discrimination	In the workplace, there is a culture that does not tolerate discrimination that infringes on human rights.
Sustainable work efficiency	Whether efficient work practices and the reduction of excessive working hours promote work–life balance	In the workplace, there is a culture of promoting work efficiency and a well-balanced way of working.
B. Employee well-being		
Overall well-being	A composite evaluation of life satisfaction and positive emotions over the past two weeks	I am satisfied with my life. I have felt excited these past two weeks.
Workplace well-being	An evaluation of a happy workplace based on a safe and secure culture, an atmosphere of a trusted workplace, an atmosphere that encourages challenge, and the degree of workplace recommendation	My workplace has a good atmosphere. I have a good working relationship with my coworkers. We encourage trying new things at my workplace. I want to recommend my workplace to others.

“[Masked term]” in the examples of items is a company-specific term related to environmental initiatives and was anonymized in this article to protect the company’s identity.

#### Financial records, and branch-level background information

We used the profit target achievement rate of each branch provided by Company A (with values below 100% indicating that the target was not met) and operating profit per employee. In addition, the number of employees and the average employee tenure in each branch were constructed as control variables.

### Statistical analyses

The analysis was conducted following the process outlined below. First, descriptive statistics and correlation coefficients for all variables were examined. Second, using the 2022 and 2023 data, in which individual employee ratings of leader behaviors were available, a confirmatory factor analysis (CFA) was conducted to assess whether the nine aspects of leader behavior defined by Company A represented distinct factors. Third, hierarchical linear modeling (HLM) was conducted to examine the relationships between branch leaders’ behaviors and branch-level outcomes. Then, based on the cross-sectional results, we identified candidate leader behaviors that enhance employee well-being and financial performance. In these models, the dependent variables were the branch-level averages of overall well-being and workplace well-being, as well as the profit target achievement rate of each branch, while the independent variables were the branch-level averages of the nine leader behaviors. All models included data collection year as a grouping variable, and the branch’s housing product segment (single-family or multifamily housing division), number of employees, and average employee tenure as control variables. In the analyses of overall well-being and workplace well-being, operating profit per employee was additionally controlled. Fourth, a cross-lagged panel model (CLPM) was conducted using four years of data to identify leader behaviors that contribute to sustained improvements in employee well-being and financial performance. We then tested whether the behaviors that showed positive effects in the HLM analyses predicted the following year’s overall well-being, workplace well-being, and profit target achievement rate. The CLPM analyses included the same control variables as the HLM analyses. All analyses were conducted using Python 3.13.3. Cronbach’s α coefficients were calculated using the *cronbach_alpha* function from *Pingouin*, correlation coefficients were calculated with the *stats.pearsonr* function in SciPy, CFA and CLPM were performed with *semopy*, and HLM was estimated with the *mixedlm* module of *statsmodels*. All regression coefficients reported in HLM and CLPM are standardized.

Prior to the main analyses, to ensure the appropriateness of aggregating individual responses to the branch level, intraclass correlation coefficients, ICC(1) and ICC(2), were calculated using a one-way ANOVA-based approach. These analyses were conducted for all variables using available individual-level data (for leader behavior variables, data were available only for 2022–2023). In these analyses, ICC(1) ranged from 0.014 to 0.058, and ICC(2) ranged from 0.56 to 0.83, indicating acceptable between-branch variability and reliability of aggregated means.

For the interpretation of the results reported below, it should be noted that correlation coefficients (*r*) are interpreted based on conventional benchmarks, whereas standardized regression coefficients (*β*) should not be interpreted in absolute terms but rather in relation to other variables within the same model. This is because *β* represents partial effects controlling for other predictors.

## Results

### Descriptive statistics and correlation coefficients

Table 3 presents the descriptive statistics and correlation coefficients for the variables used in this study to examine the basic characteristics of the data and the simple relationships among the variables, in which each branch in each year was treated as an independent sample. Initially, positive correlations were observed among the nine ratings of leader behaviors (*rs* = 0.559 to 0.869, *ps* < 0.000). These ratings were also positively correlated with overall well-being and workplace well-being (*rs* = 0.164 to 0.543, *ps* < 0.001). In contrast, none of the leader behaviors showed a positive correlation with the profit target achievement rate or operating profit per employee. Instead, corporate strategy and environmental initiatives showed negative correlations with these financial indicators (*rs* = –0.157 to –0.320, *ps* < 0.002). Furthermore, corporate strategy, environmental initiatives, and workplace strategy were negatively correlated with the number of employees in each branch (*rs* = –0.102 to –0.292, *ps* < 0.040), whereas corporate strategy, environmental initiatives, and legal compliance were positively correlated with average employee tenure (*rs* = 0.108 to 0.156, *ps* < 0.030).

Correlations were also observed among variables other than leader behaviors. Overall well-being and workplace well-being showed a strong positive correlation (*r* = .822, *p* < .000), and the profit target achievement rate and operating profit per employee also showed a strong positive correlation (*r* = .956, *p* < .000). In addition, overall well-being was positively correlated with the profit target achievement rate and operating profit per employee (*rs* = 0.127 to 0.133, *ps* < 0.010). Furthermore, the number of employees in each branch was negatively correlated with overall well-being and workplace well-being (*rs* = –0.106 to –0.157, *ps* < 0.033), whereas it was positively correlated with the profit target achievement rate and operating profit per employee (*rs* = 0.325 to 0.368, *ps* < 0.000). By contrast, average employee tenure was positively correlated with overall well-being and workplace well-being (*rs* = 0.174 to 0.182, *ps* < 0.000), whereas it was negatively correlated with the profit target achievement rate and operating profit per employee (*rs* = –0.228 to –0.246, *ps* < 0.000).

**Table 3:** Descriptive statistics and correlations.

	Category	Variables	Average	*SD*	1	2	3	4	5	6	7	8	9	10	11	12	13	14
1	Leader behaviors	Corporate strategy	79.00	5.21	–													
2		Environmental initiatives	80.74	4.92	0.789***	–												
3		Workplace strategy	81.20	4.84	0.845***	0.703***	–											
4		Communication	77.31	5.73	0.678***	0.645***	0.713***	–										
5		Employee development	77.49	4.93	0.724***	0.615***	0.817***	0.775***	–									
6		Diversity	78.69	4.87	0.651***	0.559***	0.755***	0.753***	0.852***	–								
7		Legal compliance	83.19	4.26	0.731***	0.646***	0.802***	0.810***	0.857***	0.817***	–							
8		Human rights	82.34	4.49	0.670***	0.576***	0.743***	0.795***	0.826***	0.811***	0.869***	–						
9		Sustainable work efficiency	76.87	6.32	0.646***	0.614***	0.706***	0.841***	0.755***	0.768***	0.787***	0.754***	–					
10	Employee well–being	Overall well-being	67.48	1.80	0.182***	0.164***	0.257***	0.282***	0.390***	0.353***	0.303***	0.239***	0.282***	–				
11		Workplace well-being	68.81	3.73	0.301***	0.288***	0.382***	0.464***	0.543***	0.455***	0.489***	0.434***	0.429***	0.822***	–			
12	Financial records	Standardized profit target achievement rate	0.00	1.00	− 0.157**	− 0.320***	0.037	− 0.088	0.063	0.068	− 0.041	− 0.015	− 0.083	0.133**	0.030	–		
13		Standardized operating profit per employee	0.00	1.00	− 0.167***	− 0.284***	0.037	− 0.078	0.060	0.097	− 0.042	− 0.009	− 0.045	0.127*	− 0.001	0.956***	–	
14	Branch-level background information	The number of employees in each branch	104.41	35.77	− 0.260***	− 0.292***	− 0.102*	− 0.088	− 0.095	− 0.010	− 0.094	− 0.015	− 0.055	− 0.106*	− 0.157**	0.325***	0.368***	–
15		Average employee tenure in each branch	14.28	1.50	0.141**	0.156**	0.044	0.040	0.045	0.016	0.108*	0.056	0.031	0.182***	0.174***	− 0.228***	− 0.246***	− 0.303***

*p < .050, **p < .010, ***p < .001. Sample size *(n)* for each correlation ranged from 398 to 408. Since the profit target achievement rate and the operating profit per employee are confidential information, z-standardized values were used.

### Factor structure of leader behaviors.

**Fig. 1:**
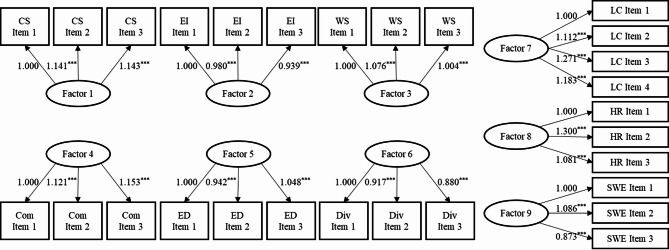
Path diagram of the CFA model, *** *p* < .001. CS = corporate strategy; EI = environmental initiatives; WS = workplace strategy; Com = communication; ED = employee development; Div = diversity; LC = legal compliance; HR = human rights; SWE = sustainable work efficiency.

Given that Company A developed the leader behavior items based on nine conceptual domains, we conducted a CFA to test the validity of the factor structure. In this analysis, to ensure a sufficient sample size for estimation, available individual-level data from 2022 to 2023 were used. Based on these data, the nine-factor model proposed by Company A was tested using responses from 17,677 individuals without missing values on the leader behavior ratings. Loadings were specified only for the items designated to each factor, with one loading fixed at 1.000 for identification, and the other loadings estimated using full information maximum likelihood (FIML). The estimation was performed using *semopy*, a library for structural equation modeling. The analysis yielded *χ²*(314) = 15,736 (*p* < .001), CFI = 0.965, TLI = 0.958, and RMSEA = 0.053. Taken together, the results indicated that the model achieved an acceptable fit. Although the χ² was significant, this is due to its sensitivity to sample size. In contrast, the CFI, TLI, and RMSEA met the criteria. Furthermore, all item loadings were significant with values above 0.87 (Fig. 1). However, the inter-factor correlations were overall high (*rs* = 0.624 to 0.877, *ps* < 0.001) (Table 4). Thresholds of 0.800 and 0.850 have been proposed for evaluating discriminant validity^31^, and in the present data three of the 36 factor pairs exceeded the 0.850 threshold, whereas ten exceeded the 0.800 threshold. Thus, the results suggested that although the nine-factor model was overall supported, several factor pairs showed conceptual overlap.

**Table 4:** Inter-factor correlations.

	Factors	1	2	3	4	5	6	7	8
1	Factor 1: corporate strategy	–							
2	Factor 2: environmental initiatives	0.826***	–						
3	Factor 3: workplace strategy	0.828***	0.837***	–					
4	Factor 4: communication	0.680***	0.673***	0.720***	–				
5	Factor 5: employee development	0.695***	0.679***	0.734***	0.877***	–			
6	Factor 6: diversity	0.686***	0.702***	0.729***	0.854***	0.833***	–		
7	Factor 7: legal compliance	0.689***	0.708***	0.737***	0.796***	0.792***	0.803***	–	
8	Factor 8: human rights	0.624***	0.652***	0.665***	0.780***	0.743***	0.831***	0.840***	–
9	Factor 9: sustainable work efficiency	0.654***	0.668***	0.710***	0.784***	0.773***	0.832***	0.784***	0.771***

***p < .001.

### Effects controlling for year and leader behavior covariation

Next, to identify leader behaviors that enhance branch-level employee well-being and the profit target achievement rate while controlling for data collection year and covariation among leader behaviors, we conducted an HLM analysis. Data collection year was specified as the grouping variable. Although the CFA indicated concerns about discriminant validity, the purpose of this study was to comparatively assess the effects of fine-grained leader behaviors on outcomes. Considering that correlation analyses also revealed different patterns of relationships with the profit target achievement rate across certain leader behaviors, the nine domains were treated as separate variables. In this analysis, the nine leader behaviors were simultaneously included in a single model to examine the independent effect of each behavior under the assumption that the other behaviors were held constant. Notably, several leader behaviors are conceptually similar and correlated with each other, which makes it difficult to distinguish their individual associations with outcomes based on correlations alone. By including them simultaneously in a single model, each coefficient represents the association of a behavior after accounting for the others. As a result, coefficients may become smaller than simple correlations, lose statistical significance, or change sign. These patterns reflect the isolation of each behavior’s unique contribution. The analysis was conducted using the *mixedlm* module in the *statsmodels* library, and coefficients were estimated by maximum likelihood (ML).

For overall well-being and workplace well-being, the model was specified as shown in Eq. (1) and the effects of each variable were estimated. In this model, the dependent variable was the branch-level mean of overall well-being or workplace well-being for branch *i* in year *j*. The explanatory variables were the branch-level means of ratings on the nine leader behaviors, denoted as $\:{X}_{k,i,j}$ (*k* = 1–9). The control variables, denoted as $\:{C}_{m,i,j}$ (*m* =1–4), included the housing product segment of the branch, the number of employees, the average employee tenure, and operating profit per employee. In this model, the intercept and the effects of leader behaviors were partitioned into a fixed intercept ($\:{\beta\:}_{0}$), fixed effects ($\:{\beta\:}_{k}$), a random intercept ($\:{u}_{0,j}$), and random effects ($\:{u}_{k,j}$). This specification allowed the intercept and the effects to vary across years, while modeling this variation separately to examine the effects of each behavior. By contrast, for the control variables $\:{C}_{m,i,j}$, only fixed effects were specified to reduce the number of estimated parameters. The term $\:{\epsilon\:}_{i,j}$ represents the error term, capturing residual variation unexplained by the model.

1 $$\:{Outcome}_{i,j}\:=\:{\beta\:}_{0}+\:{u}_{0,j}+\:{\sum\:}_{k=1}^{9}\mathrm{(\:}{\beta\:}_{k}+\:{u}_{k,j}\mathrm{)}{\:X}_{k,\:i,\:j}\:+\:\sum\:_{m=1}^{4}{\gamma\:}_{m}{C}_{m,i,j}+\:{\epsilon\:}_{i,j}$$

In contrast, for the profit target achievement rate, the model was specified as shown in Eq. (2), and the effects of each variable were estimated. The basic structure of the model was the same as that of Eq. (1), except that operating profit per employee was not included as a control variable.

2 $$\:{Outcome}_{i,j}\:=\:{\beta\:}_{0}+\:{u}_{0,j}+\:{\sum\:}_{k=1}^{9}\mathrm{(\:}{\beta\:}_{k}+\:{u}_{k,j}\mathrm{)}{\:X}_{k,\:i,\:j}\:+\:\sum\:_{m=1}^{3}{\gamma\:}_{m}{C}_{m,i,j}+\:{\epsilon\:}_{i,j}$$

Before examining the effects of leader behaviors, multicollinearity among the fixed-effect predictors was assessed, given the relatively high correlations among leader behavior variables indicated by the CFA. Variance inflation factors (VIFs) were calculated using ordinary least squares (OLS) models including all explanatory variables specified in Eqs. (1) and (2), respectively. Because multicollinearity is determined by the correlation structure among predictors, this approach provides an appropriate diagnostic for the HLM analyses. Although some VIF values were relatively high, all values were below 10, a commonly used rule-of-thumb threshold. This suggests that severe multicollinearity was not present. The VIF values are reported in Table 5.

Table 6 presents all estimation results. First, ratings of employee development showed significant positive fixed effects on overall well-being (*β* = 0.370, 95% CI 0.146 to 0.595, SE = 0.114, *p* = .001) and workplace well-being (*β* = 0.341, 95% CI 0.040 to 0.642, SE = 0.154, *p* = .056). In addition, ratings of communication showed marginally significant positive fixed effects on overall well-being (*β* = 0.240, 95% CI − 0.028 to 0.509, SE = 0.137, *p* = .080) and workplace well-being (*β* = 0.315, 95% CI − 0.007 to 0.637, SE = 0.164, *p* = .056). Furthermore, ratings of sustainable work efficiency showed a significant positive fixed effect on workplace well-being (*β* = 0.234, 95% CI 0.042 to 0.426, SE = 0.098, *p* = .017). By contrast, ratings of corporate strategy showed negative fixed effects on overall well-being (*β* = −0.195, 95% CI − 0.415 to 0.025, SE = 0.112, *p* = .082) and workplace well-being (*β* = −0.240, 95% CI − 0.435 to − 0.045, SE = 0.099, *p* = .016), while ratings of human rights also showed significant negative fixed effects on overall well-being (*β* = −0.388, 95% CI − 0.582 to − 0.194, SE = 0.099, *p* < .001) and workplace well-being (*β* = −0.262, 95% CI − 0.445 to − 0.079, SE = 0.093, *p* = .005). Finally, only ratings of workplace strategy showed a significant positive fixed effect on the profit target achievement rate (*β* = 0.308, 95% CI 0.019 to 0.597, SE = 0.147, *p* = .037), and no leader behaviors showed negative fixed effects.

**Table 5:** VIFs for fixed-effect predictors in Eqs. (1) and (2).

Explanatory variables in the model	VIF	
	Equation (1)	Equation (2)
Corporate strategy (*β*_*1*_)	5.122	5.170
Environmental initiatives (*β*_*2*_)	3.546	3.527
Workplace strategy (*β*_*3*_)	6.007	5.933
Communication (*β*_*4*_)	4.736	4.729
Employee development (*β*_*5*_)	6.317	6.170
Diversity (*β*_*6*_)	5.099	5.126
Legal compliance (*β*_*7*_)	6.669	7.051
Human rights (*β*_*8*_)	5.273	5.365
Sustainable work efficiency (*β*_*9*_)	4.340	4.371
Housing product segment (single-family: 1, multifamily: 2) (*γ*_*1*_)	2.380	2.125
The number of employees (*γ*_*2*_)	1.511	1.477
Average employee tenure (*γ*_*3*_)	1.222	1.231
Operating profit per employee (*γ*_*4*_)	1.669	-

VIF = variance inflation factor. VIFs were calculated based on OLS models including all explanatory variables in each equation. Values below 10 are commonly interpreted as indicating no severe multicollinearity.

**Table 6:** HLM results for effects of leader behaviors on outcomes.

	Effects on overall well-being			Effects on workplace well-being			Effects on profit target achievement rate		
	β	95% CI	SE	β	95% CI	SE	β	95% CI	SE
Fixed effects									
Intercept ($\:{\beta\:}_{0})$	0.033	[– 0.168, 0.234]	0.103	0.174*	[0.022, 0.326]	0.077	0.039	[– 0.349, 0.427]	0.198
Corporate strategy ($\:{\beta\:}_{1}$)	– 0.195†	[– 0.415, 0.025]	0.112	– 0.240*	[– 0.435, – 0.045]	0.099	– 0.160	[– 0.419, 0.100]	0.132
Environmental initiatives ($\:{\beta\:}_{2}$)	– 0.032	[– 0.248, 0.183]	0.110	– 0.130	[– 0.338, 0.078]	0.106	– 0.080	[– 0.399, 0.239]	0.163
Workplace strategy ($\:{\beta\:}_{3}$)	– 0.005	[– 0.239, 0.229]	0.120	0.033	[– 0.18, 0.247]	0.109	0.308*	[0.019, 0.597]	0.147
Communication ($\:{\beta\:}_{4}$)	0.240†	[– 0.028, 0.509]	0.137	0.315†	[– 0.007, 0.637]	0.164	0.194	[– 0.060, 0.448]	0.130
Employee development ($\:{\beta\:}_{5}$)	0.370**	[0.146, 0.595]	0.114	0.341*	[0.04, 0.642]	0.154	0.182	[– 0.116, 0.481]	0.152
Diversity ($\:{\beta\:}_{6}$)	0.189	[– 0.056, 0.435]	0.125	0.027	[– 0.173, 0.228]	0.102	– 0.071	[– 0.356, 0.214]	0.145
Legal compliance ($\:{\beta\:}_{7}$)	0.001	[– 0.254, 0.257]	0.130	0.116	[– 0.08, 0.312]	0.100	– 0.098	[– 0.395, 0.199]	0.152
Human rights ($\:{\beta\:}_{8}$)	– 0.388***	[– 0.582, – 0.194]	0.099	– 0.262*	[– 0.445, – 0.079]	0.093	– 0.125	[– 0.369, 0.119]	0.125
Sustainable work efficiency ($\:{\beta\:}_{9}$)	0.166	[– 0.036, 0.367]	0.103	0.234*	[0.042, 0.426]	0.098	– 0.115	[– 0.312, 0.081]	0.100
Housing product segment (single-family: 1, multifamily: 2) ($\:{\gamma\:}_{1}$)	– 0.200**	[– 0.339, – 0.061]	0.071	– 0.330***	[– 0.458, – 0.202]	0.065	0.336***	[0.216, 0.457]	0.062
The number of employees ($\:{\gamma\:}_{2}$)	– 0.025	[– 0.125, 0.076]	0.051	– 0.026	[– 0.113, 0.061]	0.044	0.113*	[0.022, 0.203]	0.046
Average employee tenure ($\:{\gamma\:}_{3}$)	0.148**	[0.059, 0.237]	0.045	0.071†	[– 0.001, 0.143]	0.037	– 0.077^†^	[– 0.163, 0.008]	0.044
Operating profit per employee ($\:{\gamma\:}_{4}$)	0.152**	[0.044, 0.261]	0.055	0.072	[– 0.025, 0.169]	0.050	–	–	–
Random effects									
Intercept (Var ($\:{u}_{0,j}$))	0.021			0.001			0.149		
Corporate strategy (Var ($\:{u}_{1,j}$))	0.007			0.008			0.037		
Environmental initiatives (Var ($\:{u}_{2,j}$))	0.002			0.008			0.077		
Workplace strategy (Var ($\:{u}_{3,j}$))	0.011			0.009			0.050		
Communication (Var ($\:{u}_{4,j}$))	0.004			0.059			0.024		
Employee development (Var ($\:{u}_{5,j}$))	0.008			0.043			0.049		
Diversity (Var ($\:{u}_{6,j}$))	0.010			0.004			0.053		
Legal compliance (Var ($\:{u}_{7,j}$))	0.011			0.003			0.046		
Human rights (Var ($\:{u}_{8,j}$))	0.000			0.003			0.036		
Sustainable work efficiency (Var ($\:{u}_{9,j}$))	0.003			0.001			0.032		
Residual (Var ($\:{\epsilon\:}_{i,j}$))	0.683			0.503			0.552		

^†^*p* < .100 *p < .050 **p < .010 ***p < .001, β = standardized regression coefficient, CI = confidence interval, SE = standard error.

### Pathways of influence on future outcomes

To examine whether leader behaviors have sustained promotive effects over time and to clarify their influence processes, we conducted CLPM analyses using data from four time points treated separately. These analyses focused on communication, employee development, sustainable work efficiency, and workplace strategy, which had shown positive effects in the HLM analyses. Control variables were specified in the same way as in the HLM analyses. Specifically, in all models, the housing product segment of the branch, the number of employees, and the average employee tenure in each year were controlled. For models with overall well-being or workplace well-being, operating profit per employee in each year was additionally controlled for. All analyses were conducted using the *semopy* package, and regression coefficients were estimated by FIML.

**Fig. 2:**
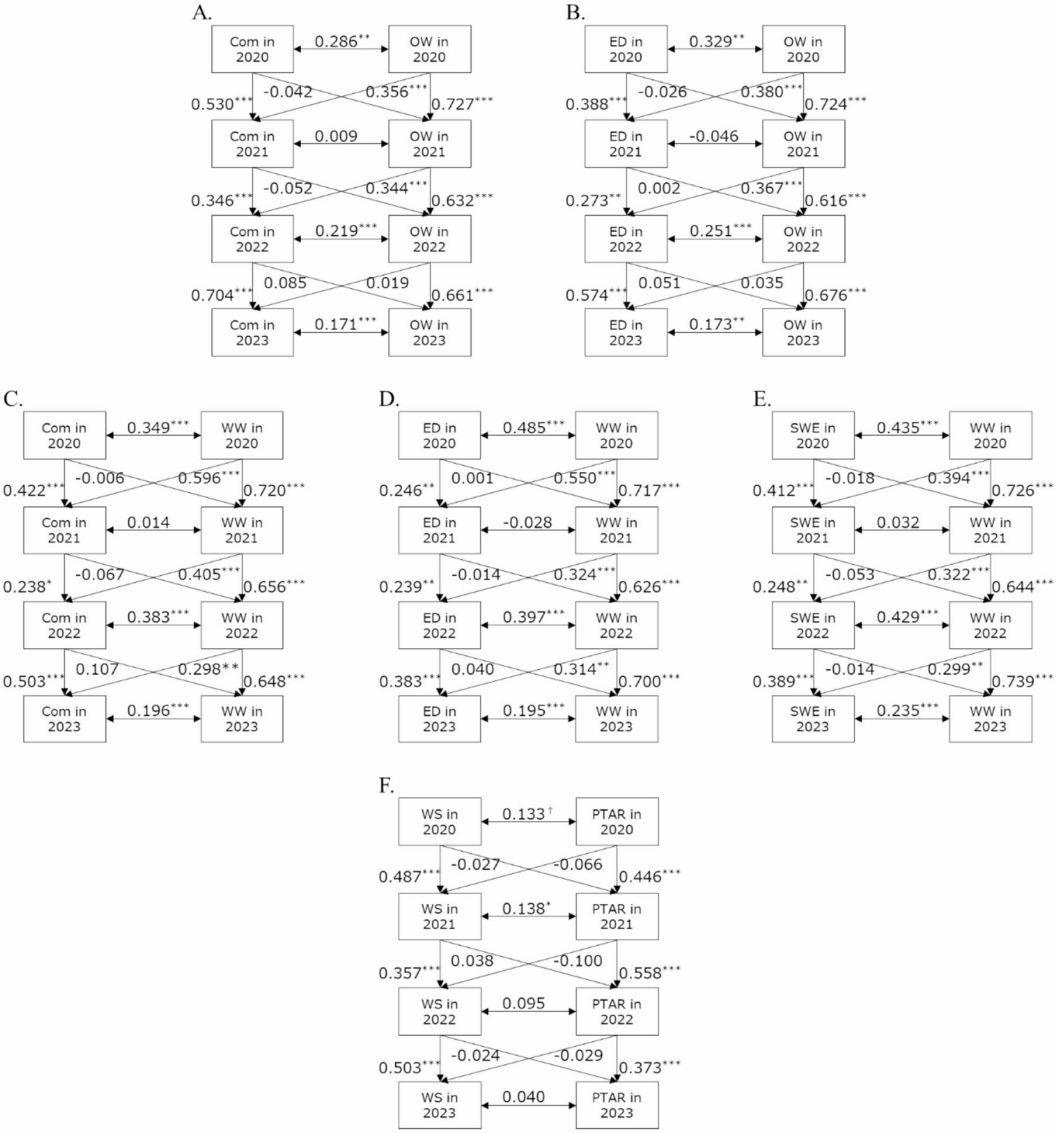
CLPM results for leader behaviors with positive effects in HLM. (**A**, **B**) overall well-being models, (**C**–**E**) workplace well-being models, and (**F**) profit target achievement rate model. Each number indicates standardized regression coefficients. Paths from control variables are omitted. ^†^*p* < .100 * *p* < .050 ** *p* < .010 *** *p* < .001. Com = communication; ED = employee development; SWE = sustainable work efficiency; WS = workplace strategy; OW = overall well-being; WW = workplace well-being; PTAR = profit target achievement rate.

First, we tested two models of overall well-being that incorporated communication and employee development as promoting factors of leader behaviors (Fig. 2A, B). In these models, overall well-being and one promotive factor in 2021, 2022, and 2023 were treated as dependent variables, while the same variables from the previous year (2020, 2021, and 2022), together with control variables, were treated as independent variables. Autoregressive and cross-lagged effects from the prior year were then estimated. The results indicated that in both models, positive autoregressive effects from the same variable in the prior year were consistently significant at all time points (*βs* = 0.273 to 0.727, *ps* < 0.010). Furthermore, in 2020, 2022, and 2023, significant positive correlations were observed between communication and overall well-being, as well as between employee development and overall well-being (*rs* = 0.171 to 0.329, *ps* < 0.010). However, the cross-lagged effects were limited. Several significant positive effects were found from overall well-being to communication and to employee development in the following year (*βs* = 0.344 to 0.380, *ps* < 0.001). In contrast, no significant effects were observed from communication or employee development to overall well-being in the subsequent year.

Next, we tested three models of workplace well-being that incorporated communication, employee development, and sustainable work efficiency as promoting factors, and the results were largely consistent with those for overall well-being (Fig. 2C–E). As with the analyses of overall well-being, positive autoregressive effects from the prior year were consistently significant at all time points in all models (*β*s = 0.238 to 0.739, *p*s < 0.050). Furthermore, in 2020, 2022, and 2023, significant positive correlations were observed between overall well-being and communication, employee development, and sustainable work efficiency (*r*s = 0.195 to 0.485, *p*s < 0.001). Regarding cross-lagged effects, only significant positive effects from workplace well-being to the promoting factors in the following year were observed (*βs* = 0.298 to 0.596, *ps* < 0.010).

Finally, we tested a model of profit target achievement rate and workplace strategy (Fig. 2F). The model design was the same as in the previous analyses, but the control variables were limited to the housing product segment of the branch, the number of employees in each year, and average employee tenure. The estimation results showed that positive autoregressive effects from the prior year were consistently significant at all time points (*β*s = 0.357 to 0.558, *p*s < 0.001). In 2020 and 2021, significant or marginally significant positive correlations were observed between workplace strategy and profit target achievement rate (*r*s = 0.133 to 0.138, *p*s < 0.100). However, no significant cross-lagged effects were observed.

## Discussion

This study used four-year panel data from a Japanese company to examine how leader behaviors relate to employee well-being, profit target achievement, and their longitudinal dynamics. In HLM controlling for year and covariation among leader behaviors, communication, employee development, and sustainable work efficiency showed positive effects on employee well-being, whereas corporate strategy and human rights showed negative effects. For profit target achievement rate, only workplace strategy showed a positive effect. CLPM examining the longitudinal processes showed no cross-lagged effects from leader behaviors to next-year employee well-being. Instead, consistent significant positive cross-lagged effects were observed from employee well-being to communication, employee development, and sustainable work efficiency in the following year, as well as significant positive correlations within the same year. No significant cross-lagged effects were found for profit target achievement rate.

Correlation analyses and CFA indicated not only basic relationships among the variables but also strong covariation among leader behaviors. Such strong covariation can be explained by the halo effect^32^, which is the tendency for high ratings on one dimension to spill over to others, or by the procedure of averaging ratings at the branch level, which may have smoothed out differences across dimensions. Because correlation coefficients alone cannot isolate the contribution of individual behaviors, we interpret the effects of leader behaviors by integrating the correlation structure, HLM, and CLPM results. In doing so, the coefficients from the HLM models should be interpreted not as causal effects but as the relative strength of associations between leader behaviors and outcomes, given that the other variables are included in the model.

Integrating the findings from HLM and CLPM, communication, employee development, and sustainable work efficiency emerge as leader behaviors that enhance employee well-being. These behaviors showed positive effects on employee well-being in HLM, and CLPM consistently indicated positive same-year correlations with well-being as well as cross-lagged effects from well-being to subsequent leader behaviors.

Taken together, the results suggest a reciprocal association between supportive leader behaviors and employee well-being, such that higher levels of supportive behaviors were associated with higher well-being in the short term, and higher well-being, in turn, was linked to more supportive behaviors in the following year. This pattern may reflect a reinforcing cycle in which supportive leader behaviors are associated with more favorable workplace conditions, and higher levels of employee well-being are, in turn, associated with more supportive leader behaviors in subsequent periods. Therefore, sustained efforts focused on communication, employee development, and sustainable work efficiency may play an important role in maintaining and supporting employee well-being. Moreover, assuming a possible bidirectional association between supportive leader behaviors and workplace well-being, leader behaviors and workplace environments may warrant regular assessment and continuous improvement.

It is also noteworthy that the present findings did not provide evidence for a unidirectional causal effect of leader behaviors on subsequent employee well-being. Thus, the observed pattern may be interpreted as the reciprocal relationship described above, although alternative explanations remain possible. Therefore, the underlying mechanisms should be examined further in future research. One possibility is that employees with higher well-being in a given year tend to evaluate their leaders more positively in the following year. This pattern may emerge even without corresponding changes in actual leader behaviors. Another possibility is that the true relationship was not detected because of the time scale used in the present study. The annual measurement interval may not have matched the time scale at which the effects of leader behaviors on employee well-being become visible. If data were collected at intervals better aligned with these processes, bidirectional cross-lagged effects between leader behaviors and employee well-being might be more clearly detected.

The processes through which the three leader behaviors described above enhance employee well-being can be interpreted within the frameworks of traditional leadership theories, and the Job Demands–Resources (JD-R) Theory^33^. First, employee development, which refers to behaviors that emphasize individual growth and provide effective training, is positioned as a core component of servant leadership theory^34^. Furthermore, according to the JD-R Theory, performance feedback and opportunities for growth can enhance future work engagement and organizational commitment, which in turn may lead to higher well-being. Next, communication, which refers to behaviors that create a cooperative and open workplace climate, constitutes the core of the *Maintenance* function in PM theory. Given that creating a climate in which employees can speak openly enhances psychological safety^35^, it is reasonable that such behavior contributes to higher levels of well-being. Finally, the effects of sustainable work efficiency, which refers to behaviors such as correcting long working hours and promoting work–life balance, can be interpreted, based on the JD-R Theory, as a process in which the reduction of job demands alleviates fatigue and anxiety and thereby enhances workplace well-being. Moreover, behaviors included in sustainable work efficiency are also represented in servant leadership theory as part of creating a supportive environment. These findings are consistent with existing theories.

On the other hand, HLM results indicated that only workplace strategy, which refers to behaviors that involve sharing strategies at the workplace and clarifying individual goals and roles, had a positive effect on profit target achievement rate. However, when CLPM results are considered, workplace strategy does not appear strong enough to exert a lasting effect in the following year. While it may improve financial performance in the short term, its effects were likely obscured by external factors such as market trends and economic fluctuations. To secure stable profits, it may not be sufficient for leaders to simply share directions with their subordinates. In other words, leaders must discern appropriate actions based on context. Previous research has conceptualized this ability as *contextual intelligence*^36^, which refers to the ability to recognize and interpret multiple, overlapping contextual factors and to integrate them to guide effective action. If leaders can read multiple layers of context—social, organizational, and cultural—, select the right direction, clearly communicate it, and clarify expectations, sustained improvements in profits may be achieved. Continuous investigation is also needed to identify leader behaviors that can sustainably improve financial performance.

Finally, HLM results suggested that corporate strategy and human rights, depending on how they are implemented, could undermine employee well-being. Specifically, corporate strategy, which refers to the extent to which company-wide policies are shared, may affect employee well-being through a reduction in psychological safety. Considering that organizational structures aiming to increase motivation through unattainable goals can undermine psychological safety^35^, one-way dissemination of policies in large companies may function as a form of top-down pressure. In contrast, human rights, which refers to strict responses against harassment and discrimination, initially showed a positive correlation but turned negative when other behaviors were included in the HLM model. This suggests that strict enforcement of rules, if carried out without dialogue or consideration, may increase employee inhibition and anxiety. The ethical aspects of leadership are often emphasized in prior research^37^, but the present study did not find that ethics alone improved employee well-being. Notably, these findings should not be interpreted as indicating that clarifying corporate strategy or promoting human rights protections is inherently detrimental. The estimated coefficients reflect associations given that other leader behaviors are held constant. Therefore, clarifying corporate strategy or promoting human rights does not in itself imply negative consequences for the workplace. Rather, top-down policy dissemination and overly controlling or punitive implementation, when carried out without sufficient dialogue, should be understood as potentially undermining employee well-being.

The present study provides detailed insights into leader behaviors associated with employee well-being and operating profit, although several limitations should be noted. These limitations highlight the importance of broadening research populations and refining analytical methods. First, the nine leader behaviors examined in this study were based on a company-specific framework, and their relatively high intercorrelations raise concerns about discriminant validity. While this study prioritized concrete insights by analyzing behaviors separately, future research should refine the factor structure in relation to existing theoretical frameworks. Second, causal inference remains limited. Although bidirectional relationships involving employee well-being were suggested, no clear causal effects were found for financial performance, which may be influenced by external factors. Future research should examine causal relationships using longitudinal data across multiple firms and time periods, as well as intervention-based approaches. Third, as the data were drawn from a single homebuilder in Japan, the generalizability of the findings is limited, and future studies should examine multiple firms and contexts. Finally, although the behavioral dimensions are more specific than those in prior research, they remain insufficiently concrete for direct practical application. Further research integrating complementary approaches, such as qualitative methods, would help generate more actionable insights.

Overall, this study provides empirical insights into the temporal processes of influence by examining the effects of fine-grained leadership behaviors on employee well-being and profit target achievement. Leadership behaviors associated with employee well-being included employee development, the promotion of communication, and the improvement of sustainable work efficiency. Strengthening these behaviors may help organizations foster a virtuous cycle with employee well-being. However, financial outcomes may not be improved by these behaviors alone. To improve profit target achievement, leaders may need to share direction at the workplace level and clarify subordinate roles. When allocating limited resources, these behaviors may serve as practical actions that can be implemented immediately. In addition, senior management may enhance both well-being and profit by evaluating and supporting these behaviors among leaders.

## Data Availability

The datasets analyzed during the current study are not publicly available due to a non-disclosure agreement between the providing company and the authors. The data was provided to the authors solely for the purpose of conducting this research and cannot be shared further.
